# Influence of untranslated regions on retroviral mRNA transfer and expression

**DOI:** 10.1186/1472-6750-13-35

**Published:** 2013-04-16

**Authors:** Anne Prel, Luc Sensébé, Jean-Christophe Pagès

**Affiliations:** 1 INSERM U966, Faculté de médecine, Université François Rabelais, Tours, 37000, France; 2 EFS Pyrénées-Méditerranée, Toulouse, 31432, France; 3 UMR 5273 CNRS-EFS-UPS-INSERM U1031, Toulouse, France; 4 Present address: UMR 5273 CNRS-EFS-UPS-INSERM U1031, Toulouse, France

**Keywords:** RNA, UTR, Retroviral transfer, Retroviral vectors, Transient expression

## Abstract

**Background:**

Deliberate cellular reprogramming is becoming a realistic objective in the clinic. While the origin of the target cells is critical, delivery of bioactive molecules to trigger a shift in cell-fate remains the major hurdle. To date, several strategies based either on non-integrative vectors, protein transfer or mRNA delivery have been investigated. In a recent study, a unique modification in the retroviral genome was shown to enable RNA transfer and its expression.

**Results:**

Here, we used the retroviral mRNA delivery approach to study the impact of modifying gene-flanking sequences on RNA transfer. We designed modified mRNAs for retroviral packaging and used the quantitative luciferase assay to compare mRNA expression following viral transduction of cells. Cloning the untranslated regions of the vimentin or non-muscular myosin heavy chain within transcripts improved expression and stability of the reporter gene while slightly modifying reporter-RNA retroviral delivery. We also observed that while the modified retroviral platform was the most effective for retroviral mRNA packaging, the highest expression in target cells was achieved by the addition of a non-viral UTR to mRNAs containing the packaging signal.

**Conclusions:**

Through molecular engineering we have assayed a series of constructs to improve retroviral mRNA transfer. We showed that an authentic RNA retroviral genomic platform was most efficiently transferred but that adding UTR sequences from highly expressed genes could improve expression upon transfection while having only a slight effect on expression from transferred RNA. Together, these data should contribute to the optimisation of retroviral mRNA-delivery systems that test combinations of UTRs and packaging platforms.

## Background

The transfer of non-integrative, non-DNA genetic information to trigger changes in cell-fate is a challenge in advanced therapeutics [1]. Achieving efficient transfer of active factors is crucial for controlling cell differentiation programs in various cell types including induced pluripotent stem cells (iPS) [2]. A viral approach could offer several advantages compared to potentially toxic chemical approaches, particularly for *in vivo* applications that might require repeated administration.

As mRNA could be a useful molecule to trigger cellular differentiation [2], access to a large amount of transferred mRNA is required. However, the efficiency of expression of transferred RNA may also profoundly influence the extent of the expected phenotypic changes. Under physiological conditions, cellular RNA expression is controlled by sequences flanking the translated region, the so-called 5′ and 3′ untranslated regions (UTRs) [3,4]. Investigation of the regulation of RNA translation has demonstrated that RNA biological availability generally depends on binding of cellular proteins and regulatory RNAs to UTRs [5]. Therefore, we anticipated that UTR sequences could have a direct impact on transgene expression for RNAs transferred using viral vectors. In conventional expression systems, such as commercially available ones, UTRs are not generally considered for modification. Most expression vectors rely on a favourable Kozak sequence, a heterologous intron and a ubiquitous, efficient polyadenylation signal, which makes these vectors amenable to improvement. For retroviral vectors, the addition of transcriptional regulatory elements, wPRE for example, improves polyadenylation and increases vector titers [6].

The RNA content of retroviral particles can be quite diverse. It includes specifically recruited RNAs bearing the packaging signal (psi) and a wide variety of captured, small cytoplasmic RNAs [7-10]. Any mRNA can be packaged into retroviral particles provided that a canonical psi sequence is cloned into the transcript [11-13]. More recently, solely inactivating the primer binding site has been sufficient to convert integrative retroviral vectors into RNA delivery systems [14]. For retroviruses, it was shown that incoming genomes are directed to cellular compartments poorly accessible to RNA interference [15], accordingly they could also be poorly translated. In infected cells, wild-type retrovirus genome translation is mostly supported by capped mRNAs produced upon integration. Interestingly, the presence of an internal ribosomal entry site within the retroviral genome suggests that some direct translation of the genomic RNA is achievable [16]. However, the level of this putative retroviral expression from incoming recombinant retroviral RNAs is not known. To date, no studies have addressed this question in the context of vectors.

In the present study, we aimed to engineer retrovirally packaged mRNAs. For this, we designed constructs to improve both the retroviral delivery of RNAs and their efficient expression after transfer into target cells. RNA biology has recently been renewed by the study of regulating RNAs, including miRNAs [17]. The half-life of cellular RNA depends on RNA processing by the RNA-induced silencing complex, as well as the complex process of mRNA decay. Influencing the mRNA decay machinery at the cellular level to improve the availability of RNAs in relevant cells is hardly conceivable. Moreover, drug-induced neutralisation of mRNA decay, which would favour the expression of a transferred RNA, could induce profound and detrimental dysregulation of the target cell differentiation program. Therefore, we evaluated and modulated mRNA stability by modifying the 5′ and 3′ UTR characteristics of mRNA. Because these modified RNAs were also designed to permit retroviral mobilisation, we used psi-containing RNAs.

## Results

### Engineering mRNA

MLV-based retroviral particles were used as vehicles to recruit and transfer mRNAs of interest [12]. Galla and collaborators have shown that such transfer was possible by modifying a retroviral vector, provided that the vector RNA has been disabled for reverse transcription while maintaining the packaging signal [14,18]. In these pioneer studies, the use of a Cre-based recombination system increased signal detection in retroviral-transduced cells. Because RNA stability and subsequent protein production of many mRNAs is dictated by elements in their UTRs, we designed a series of constructs with various 5′ and 3′ UTRs flanking a luciferase gene as a marker [4]. Luciferase has suitable sensitivity and a short half-life for easily detecting the effect of an improvement introduced with the transferred mRNA [19,20].

**Figure 1:**
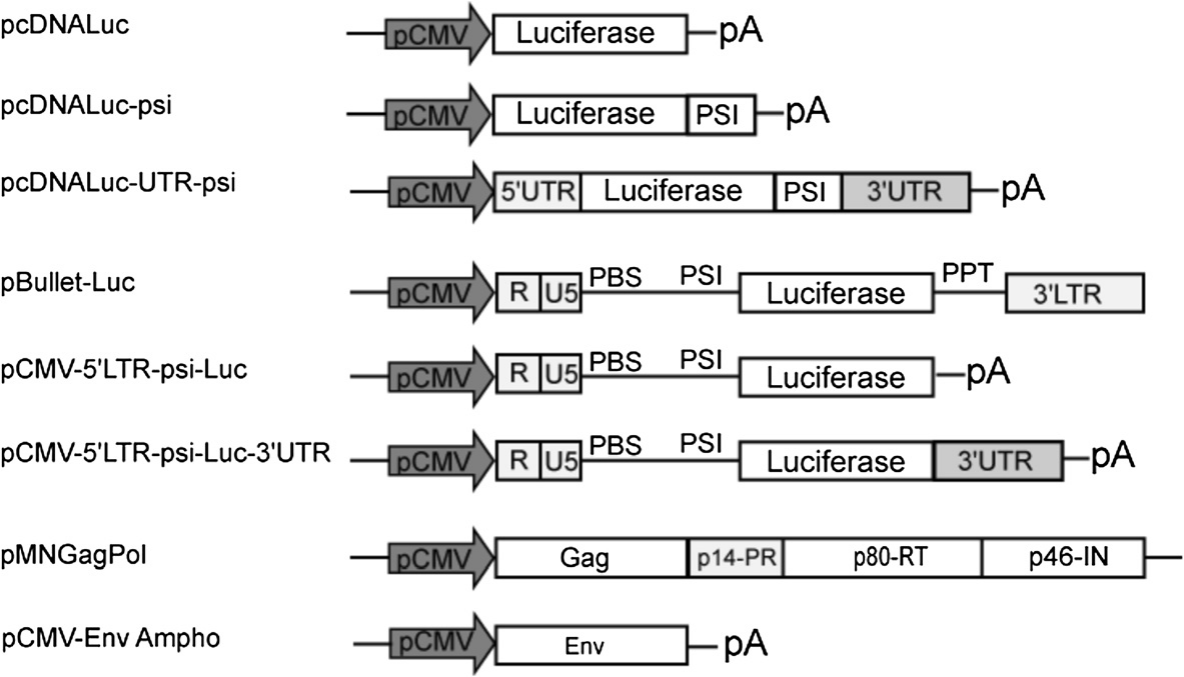
**Schematic representation of luciferase vectors used for recruitment into retroviral particles.** psi: MLV packaging signal. PBS: retroviral primer binding site. PPT: polypurine tract, priming + strand synthesis following the fist jump during reverse transcription. pA: Bovine Growth Hormone polyadenylation signal. The last two constructs (pMNGag-Pol and pCMV-Env-Ampho) were used for transcomplementation in order to drive particles formations.

We selected 5′ and 3′ UTRs from genes known to have a high level of expression such as vimentin and non-muscular myosin heavy chain (NMHC) [21,22]. β-globin or β-actin were also tested but were eliminated early in the evaluation process (data not shown). Vimentin is a protein which assembles into type III intermediate filaments and a marker of cells of mesenchymal origin (e.g., fibroblasts and myofibroblasts). Vimentin is one of the most widely expressed and highly conserved proteins of the type III IF protein family [21]. The MYH9 gene encodes a large (224 kDa) cytoplasmic myosin IIA heavy chain that contains an IQ domain (Isoleucine-Glutamine domain) and a myosin head-like domain and is involved in several important functions including cytokinesis, cell motility and maintenance of cell shape [23]. To enable the recruitment of these UTR-containing mRNAs into retroviral particles, we inserted a minimal sequence covering the packaging signal of MLV (+206 to +512, Genbank: AF033811) at the 3′ end of the reporter gene, 5′ to the tested 3′ UTR (Figure 1) [8,11,24]. Hereafter, all constructs containing the 5′, 3′ UTRs and the minimal psi flanking the luciferase gene were called pcDNA-Luc-UTR-psi or simple mRNAs.

In parallel, to promote efficient mRNA recruitment into retroviral particles, we used mRNAs from a regular retroviral vector (pBullet-Luc) that we truncated at its 3′ end to prevent reverse transcription and genomic integration [25]. We substituted the ppt and the 3′ LTR of pBullet-Luc with the 3′ UTR of vimentin or NMHC, followed by the polyA signal from the bovine growth hormone (pCMV-5′LTR-psi-Luc and pCMV-5′LTR-psi-Luc-3′UTR, see Methods). Hence, all the pBullet based constructs had the same retroviral-derived 5′ and differed one to the other only by the 3′ UTR region, and these vectors were referred as “truncated retroviral mRNAs”. The mRNA transcribed from these vectors contained optimal MLV packaging sequence (+206; +1106, Genbank: AF033811) [8,11].

We next studied retroviral packaging, using a transient MLV retroviral complementation system. The pMN complementation vector, encoding intact GAG and POL genes, was transfected together with a plasmid expressing an envelope allowing for a wide choice of target cells (Figure 1). Usually, the pantropic VSV-G envelope is used for this purpose. However, spontaneous, unspecific budding of cellular vesicles, leading to pseudoparticle formation has been described when using the VSV-G envelope [26,27]. Importantly, these VSV-G-vesicles were shown to contain various cellular components, including non-specifically mobilised cellular RNAs [28], a condition potentially leading to flawed interpretation of mobilisation data. Thus, we chose to pseudotype the particles by using the 4070A amphotropic envelope, targeting most cells of human origin (Figure 1).

### Evaluating mRNA content in transfected cells

To evaluate the effect of the UTR from vimentin or NMHC on luciferase expression, 293FT cells were transfected with the different constructs (pcDNA-Luc-psi or pcDNA-Luc-UTR-psi and pCMV-5′LTR-psi-Luc+/−3′UTR). All constructs allowed luciferase expression (See below).

**Figure 2:**
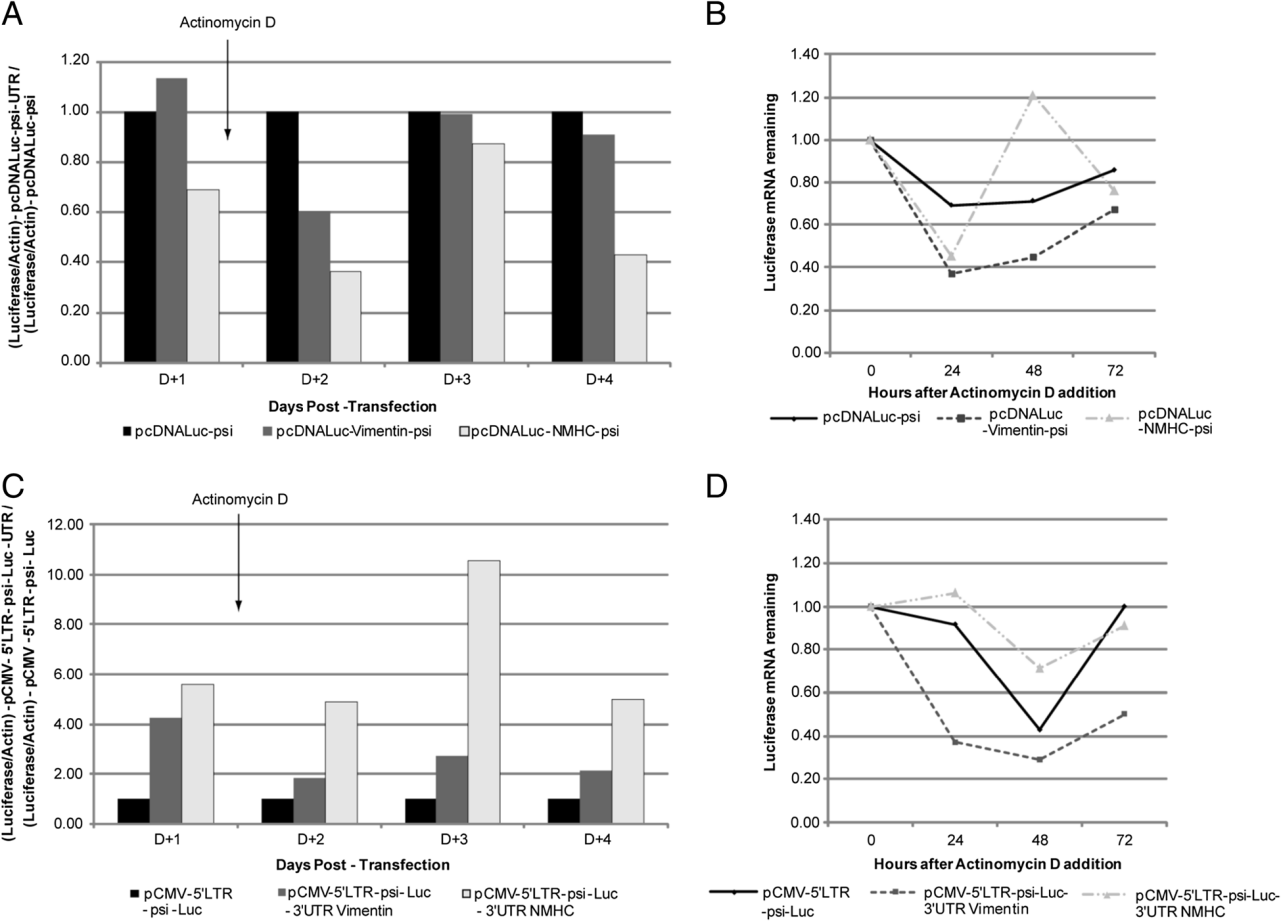
**mRNA cellular persistence: quantitative RT-PCR of Non-viral (A and B) and Truncated-retroviral (C and D) mRNA.** 293FT cells were transfected with constructs. One day after transfection, transcription was blocked by actinomycin D, and luciferase-mRNA levels were measured at 24, 48 and 72 h. Luciferase mRNA was normalized to that of actin. (**A** and **C**) Comparison of luciferase mRNA level in transfected cells, for each condition the amount was normalized to that of b-actin in cells transfected with pcDNA-Luc-psi or pCMV-5′LTR-psi-Luc and pcDNA-Luc-psi-UTR or pCMV-5′LTR-psi-Luc-UTR. The bars indicate relative amounts compared to control condition (in A pcDNA-Luc-psi and in C pCMV-5′LTR-psi-Luc). Number of experiments, N = 2. (**B** and **D**) Evolution of the specific amount of the different RNAs. RNA extracted from cells immediately after the addition of actinomycin D (time-point 0) was used to define the initial level of mRNA and arbitrarily set to 100%, at 24, 48 and 72 hours the amount of the different RNAs are compared to this initial point. Number of experiments, N = 2.

We first studied the duration of mRNA cytoplasmic availability following transcription, with the UTR-minus pcDNA-Luc-psi as a control. Quantitative RT-PCR (RT-qPCR) revealed that vimentin UTR did not impact on mRNA expression, whereas NMHC seemed to reduce it (Figure 2A; D + 1). To monitor the persistence of the RNA following transcription, cells were treated with the transcription inhibitor actinomycin D [29], at day one following transfection. The luciferase mRNA level was then measured 2 to 4 days after transfection, reflecting the half-life of the mRNA (Figure 2A). As compared with pcDNA-Luc-psi alone, pcDNA-Luc-Vimentin-psi produced the next-highest mRNA level, while NMHC UTRs were not efficient at stabilizing the mRNA level (Figure 2A). To further understand the effect of actinomycin D, we calculated the relative amount of each individual construct following transcription inhibition. Under these conditions, actinomycin D induced a decrease of all mRNA levels in transfected cells (Figure 2B). Unexpectedly, as compared with pcDNA-Luc-Vimentin-psi or pcDNA-Luc-NMHC-psi, pcDNA-Luc-psi seemed to produce more stable mRNA transcripts (Figure 2B). At day 4, luciferase mRNA levels increased in all transfected cells, due to actinomycin D inactivation or clearance.

Using the same protocol, we measured the amount of truncated mRNA in cells transfected with pCMV-5′LTR-psi-Luc or pCMV-5′LTR-psi-Luc-UTR plasmids. pCMV-5′LTR-psi-Luc gave the lowest level of mRNA expression as compared with pCMV-5′LTR-psi-Luc-3′UTR Vimentin or pCMV-5′LTR-psi-Luc-3′UTR NMHC (Figure 2C). Adding actinomycin D had no effect on the expression profile of the 3 individual plasmids (Figure 2C). For this retroviral platform, we could only observed a trend suggesting that RNA from the pCMV-5′LTR-psi-Luc- 3′UTR NMHC could be more stable than that from pCMV-5′LTR-psi-Luc or pCMV-5′LTR-psi-Luc-3′UTR Vimentin (Figure 2D).

### Evaluating modified mRNA expression

**Figure 3:**
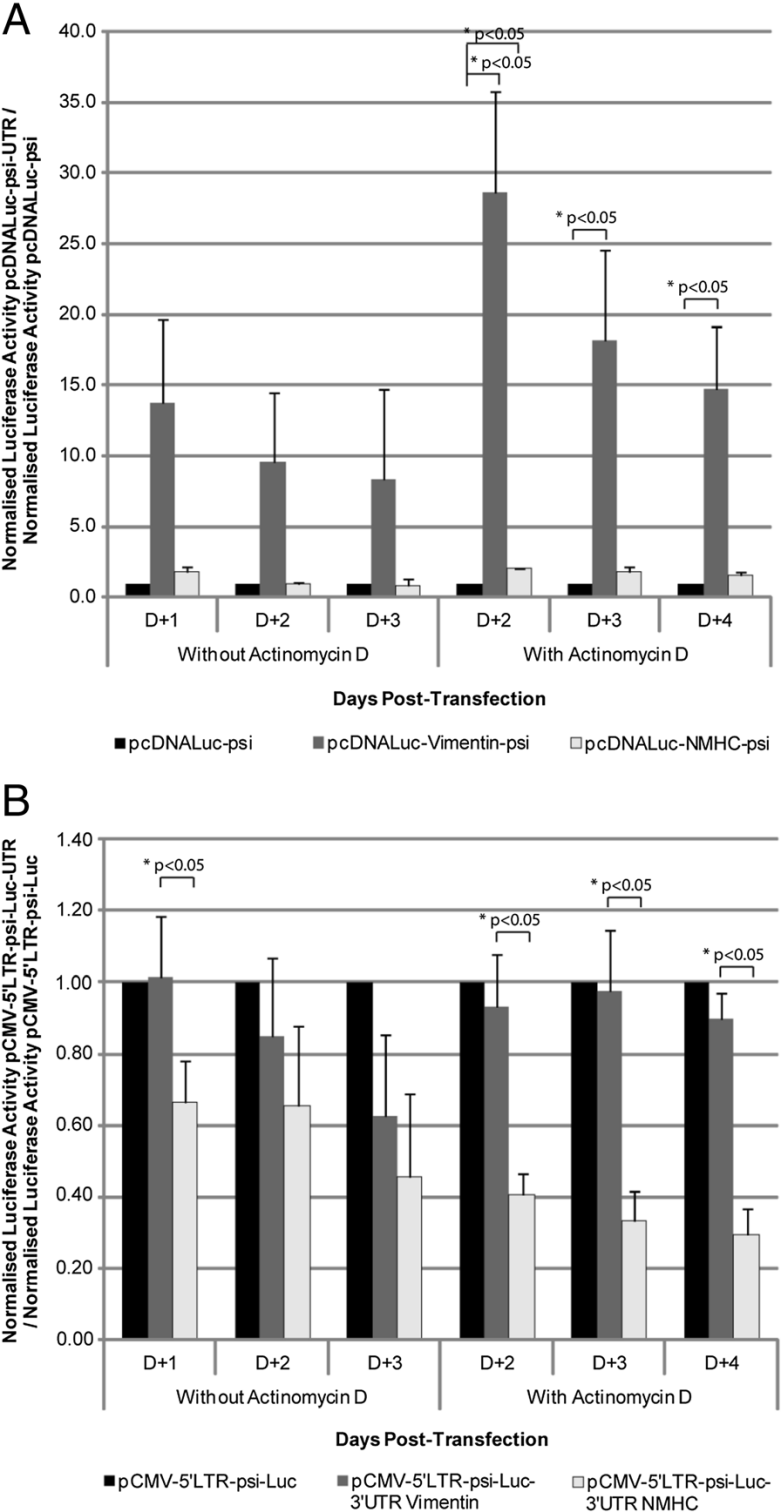
**Luciferase expression from Non-viral (A) and Truncated-retroviral (B) constructs.** 293FT cells were transfected with the different constructs. One day after transfection, transcription was blocked by actinomycin D. 2 to 4 days post-transfection, luciferase activity was measured in lysed cells. Number of experiments, N = 3. (3**A**) The analysis was performed using pcDNALuc-psi as comparator for each condition. To evaluate the significance of the observed differences, we performed a one sample *t*-test. This statistical test allowed us to determine if the means observed for pcDNA-Luc-Vimentin-psi and pcDNA-Luc-NMHC-psi were significantly different to the reference value fixed to 1, which correspond to pcDNA-Luc-psi. *p-value <0.05. (3B) As the signal from pCMV-5′LTR-psi-Luc was not different from the one from pCMV-5′LTR-psi-Luc-Vimentin, we evaluated the significance of the observed differences between pCMV-5′LTR-psi-Luc-Vimentin and pCMV-5′LTR-psi-Luc-NMHC, this was achieved with a Wilcoxon-Mann–Whitney. *p-value < 0.05.

We next evaluated the functionality of the modified mRNAs in transfected cells. At 1 to 4 days after transfection, luciferase expression was increased with pcDNA-Luc-UTR-psi plasmids containing vimentin UTR and also with NMHC UTR, but not significantly (Figure 3). To investigate any correlation of mRNA stability with a “long-term” expression of the transgene, we measured luciferase activity following actinomycin D treatment (Figure 3A). The background, putatively resulting from the accumulation of the marker protein, was considered negligible because we evaluated luciferase expression 1 day after actinomycin D exposure. This lag time significantly exceeded the time needed for the clearance of the luciferase expressed at the early time point, this protein having a 2-h half-life [30]. Again, luciferase expression was significantly increased in cells transfected with pcDNA-Luc-UTR-psi containing the vimentin UTR. Therefore, we concluded that UTR regions from vimentin improved mRNA expression, possibly through increased mRNA stability or translation.

Luciferase expression obtained with truncated retroviral mRNA, was comparable to or lower when comparing cells transfected with pCMV-5′LTR-psi-Luc to pCMV-5′LTR-psi-Luc-3′UTR Vimentin or pCMV-5′LTR-psi-Luc-3′UTR NMHC (Figure 3B). Actinomycin D did not change the profile or the ratio of the expression for each construct (Figure 3B). While the RNA levels measured seemed to be the highest (Figure 2D), pCMV-5′LTR-psi-Luc-3′UTR NMHC gave the lowest Luciferase expression (Figure 3B). This could be explained by the activity of Let-7f, a miRNA targeting NMHC 3′UTR and expressed in various cell types [31].

### Recruiting mRNA into retroviral particles

**Figure 4:**
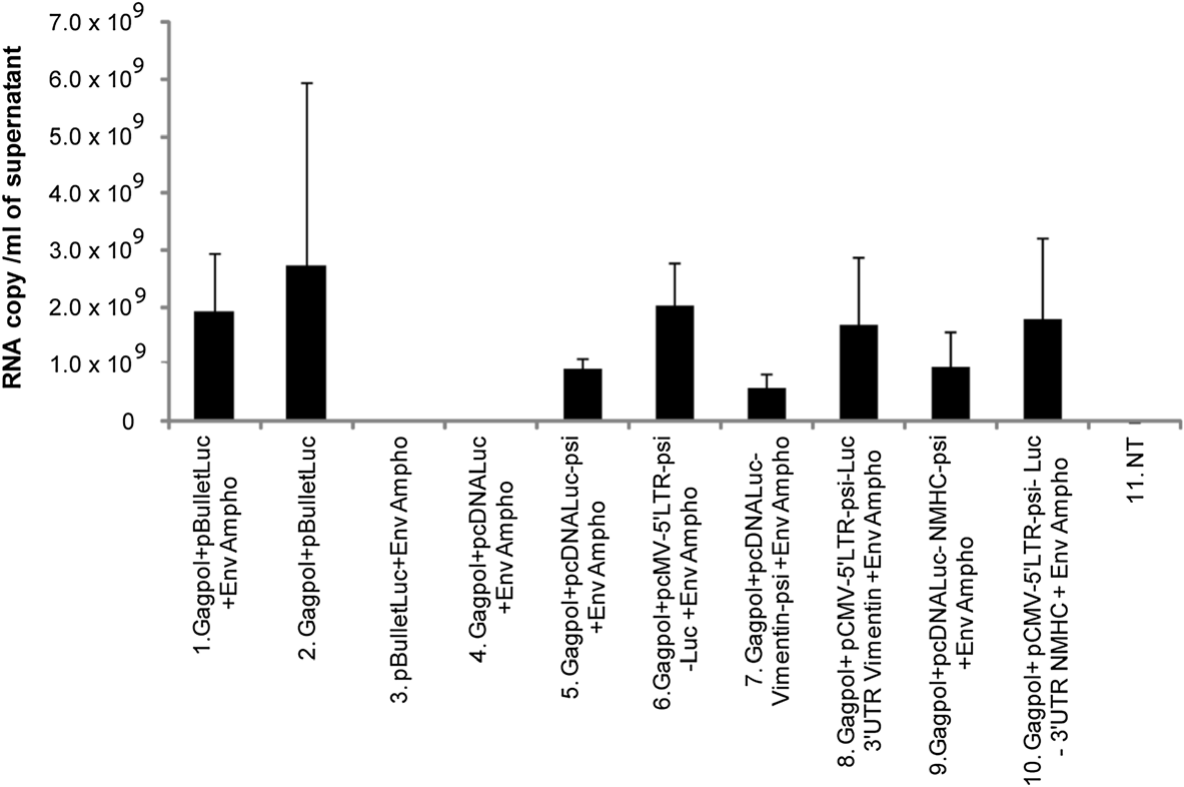
**Recruitment of the modified mRNA into retroviral particles.** Quantitative RT-PCR analysis of luciferase-mRNA content extracted from retroviral particles produced in cells transfected with the packaging constructs and the test-constructs, 48 h post-transfection. All results were normalized for transfection-efficiency with GFP expression determined by FACS analysis. Comparisons were performed using Wilcoxon-Mann–Whitney analysis. NT: untransfected cells. Number of experiments, N = 3.

To evaluate the recruitment of simple and truncated mRNA into retroviral particles, each vector was transfected with MLV packaging constructs. For each condition, a same minimal amount of a GFP expression vector was co-transfected. We harvested culture supernatants for the different vectors at 48 and 72 h after transfection, and analysed luciferase-mRNA content of retroviral particles by RT-qPCR. At 48 h post-transfection, we examined recruitment of simple and truncated mRNA into retroviral particles by RT-qPCR (Figure 4). All results were normalised according to transfection efficiencies based on GFP expression determined by FACS analysis at the time of supernatant harvest. We obtained only a faint level of packaging for pcDNA-Luc, the control expression vector for Luciferase lacking a retroviral psi, which confirmed the crucial role of a retroviral packaging signal for mobilizing mRNA into retroviral particles. As another control, RT-qPCR confirmed that the unique expression of a retroviral envelope was not associated with luciferase-mRNA packaging (Figure 4, lane 3). For psi containing mRNAs, the RT-qPCR indicated packaging of all mRNA products (Figure 4, lanes 6–10). While not statistically significant, we also noticed a clear trend, suggesting that truncated-retroviral vector-RNAs were more potent in promoting mRNA packaging (Figure 4, lanes 6,8,10), as compared with simple mRNAs (Figure 4, lanes 5,7,9). Also, within pcDNA-Luc-UTR-psi or pCMV-5′LTR-psi-Luc-UTR, results did not significantly differ from each other (Figure 4).

### Transfer study of simple and truncated mRNA into cells

**Figure 5:**
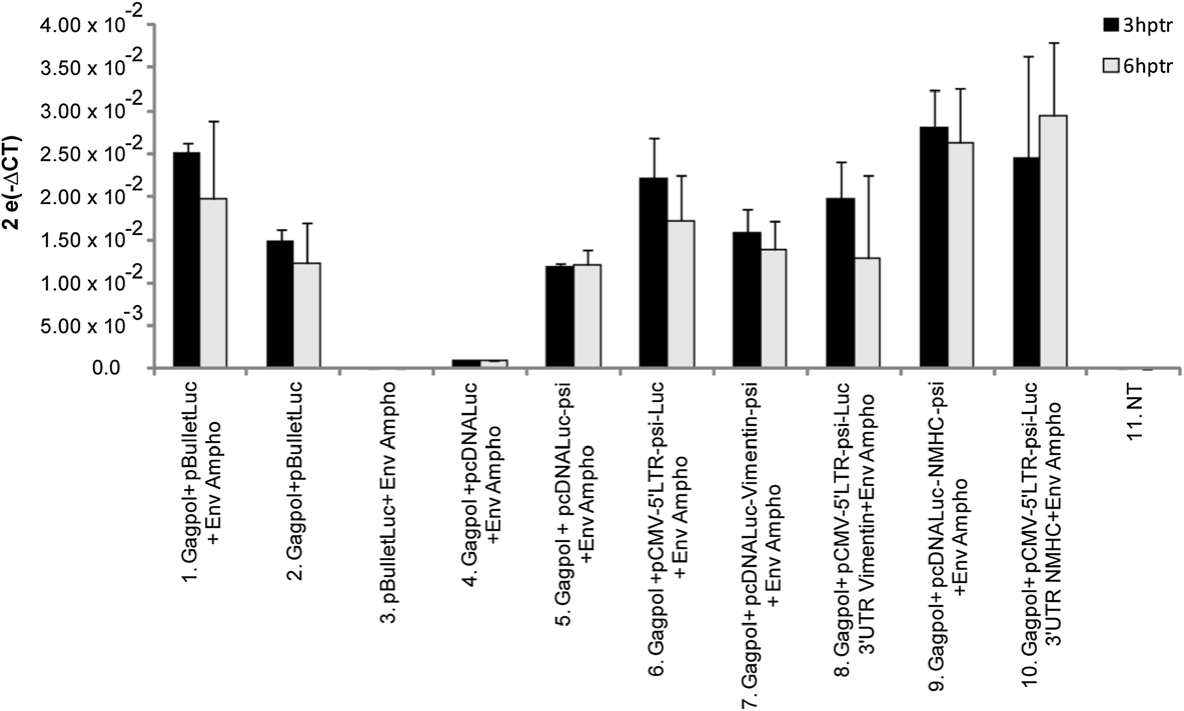
**Retroviral transfer of mRNA into 293FT cells using relative RT-qPCR analysis.** Quantitative RT-PCR analysis of luciferase mRNA levels in 293FT cells, 3 and 6 h post-transduction with retroviral particles harvested 48 h post-transfection, in each condition the luciferase mRNA was normalized to that of β-actin. Comparisons were performed using Wilcoxon-Mann–Whitney analysis, despite a tendency, no statistically significant differences could be observed. NT: untransfected cells. Number of experiments, N = 3.

**Figure 6:**
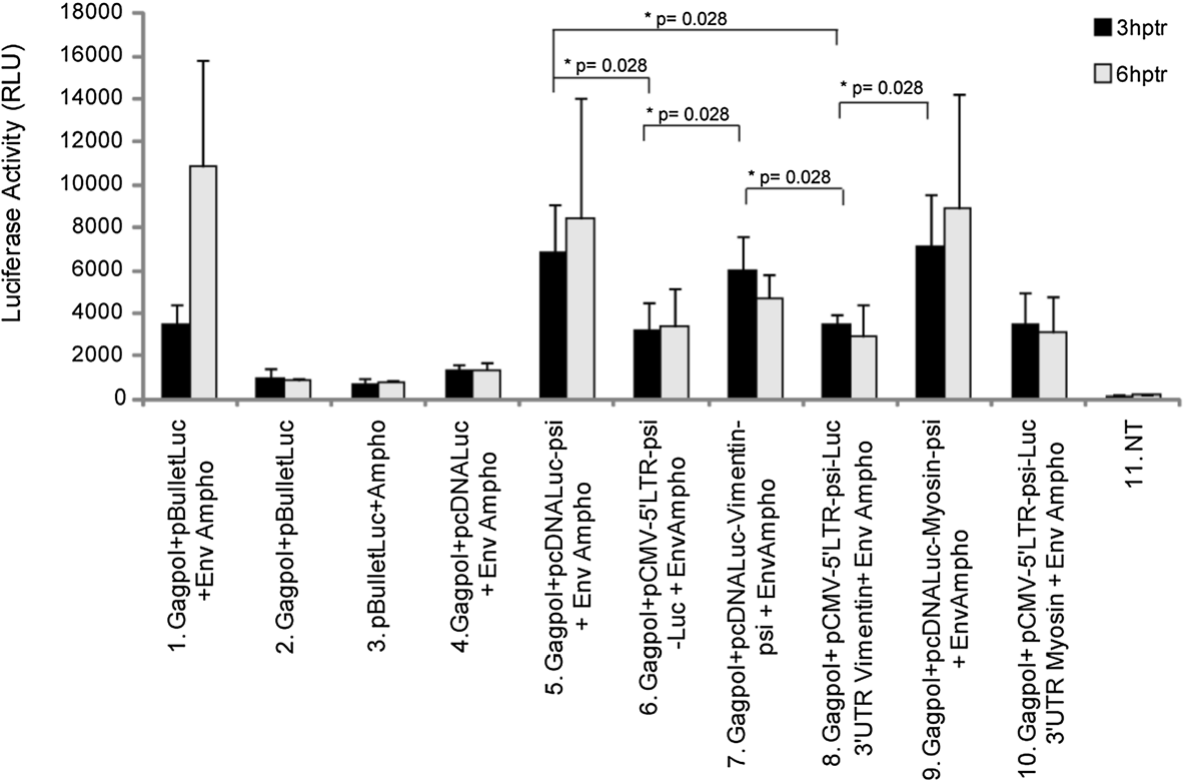
**Luciferase activity upon retroviral transfer of mRNA into 293FT cells. 293FT cells were transduced with 100 μl supernatants containing retroviral particles harvested 48 h post-transfection.** 3 or 6 h post-transduction, cells were lysed and luciferase activity was measured. We observed a statistically significant difference between the control condition 4 and all the test constructs (5 to 10) (p-value < 0,03). Statistical comparisons between the different constructs were performed on the 3 hours time-point and evaluated using a Wilcoxon-Mann–Whitney analysis. hptr, hours post-transfection. NT: untransfected cells. Number of experiments, N = 3.

To assess the retroviral transferability of the modified mRNA, we used the supernatant generated using cells co-transfected with test-vectors and MLV packaging constructs. 293FT target cells were then transduced with equivalent amount of retroviral particles harvested 48 and 72 h post-transfection. We evaluated the transfer of packaged mRNA into transduced cells by RT-qPCR as above and by measuring luciferase activity 3 and 6 h after transduction (Figures 5 and 6).

For RT-qPCR analysis of RNA delivery, all results were normalised as described above. To circumvent stability interference, the RNA transfer was studied shortly after transduction. RT-qPCR analysis revealed luciferase-mRNA in cells transduced for almost all conditions. As previously shown for mRNA recruitment, we observed a tendency indicating that the level of luciferase mRNA was slightly higher in cells transduced with retroviral particles containing truncated retroviral mRNA (pCMV-5′LTR-psi-Luc-UTR) (Figure 5, lanes 6,8,10). Moreover, the level of luciferase mRNA was increased in cells transduced with retroviral particles containing mRNA with the NMHC UTR (Figure 5; lanes 9 and 10). Finally, the luciferase mRNA level was only slightly decreased at 6 h post transduction (Figure 5; compare black and grey bars), which suggests that the retrovirally transferred mRNA harboured a certain stability following cell entry. Of note, RT-qPCR analysis revealed luciferase mRNA even in cells transduced with retroviral particles from cells producing a retroviral vector missing an envelope (Figure 5, lane 2). Thus, part of the signal observed in RNA-test conditions corresponded to retroviral particles adsorbed at the surface of transduced cells. Importantly, the signal obtained with the supernatant from particles produced without psi-containing luciferase-mRNA was almost not detectable, which confirmed the negligible contribution of non-specific packaging (Figure 5, lane 4).

We next evaluated the functionality of transduced mRNAs by a luciferase assay. 293FT cells were transduced with retroviral particles for 3 or 6 h, and then cells were lysed to allow measurement of luciferase-activity (Figure 6). Importantly, in contrast to RT-qPCR results, retroviral particles without any functional (fusogenic) envelope did not promote luciferase expression (Figures 5 and 6; compare lanes 2). This indicated that the simple adsorption of the particles was not sufficient to direct mRNA delivery. Interestingly, we observed that luciferase expression was higher in cells transduced with retroviral particles packaging pcDNA-Luc-UTR-psi mRNAs (Figure 6, lanes 5,7,9) than with pCMV-5′LTR-psi-Luc-UTR, the truncated retroviral vector RNA (Figure 6, lanes 6,8,10). Among the pcDNA-Luc-UTR-psi mRNAs, the highest expression seemed to be for pcDNA-Luc-NMHC-psi (Figure 6, lane 9). Luciferase activity remained stable between 3 and 6 h after transduction (Figure 6, compare black and grey bars), which suggested a certain stability of the mRNAs, but the 2 hours half-life of the luciferase could also partially explain this observation.

**Figure 7:**
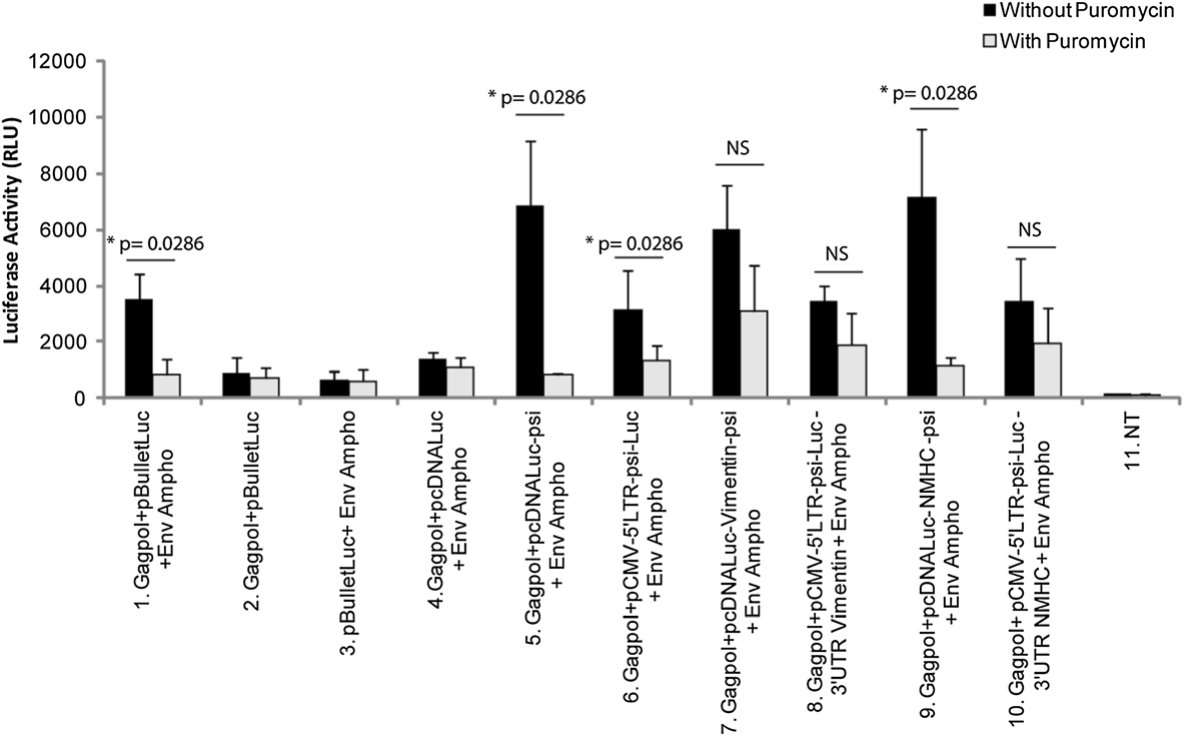
**Luciferase activity upon retroviral transfer of mRNA into 293FT cells with and without translation inhibitor.** 293FT cells were transduced with 100 μl supernatants containing retroviral-particles with or without 3 μg/ml puromycin. After 3 h, cells were lysed and luciferase activity was measured. Results were normalized by transfection efficiency with GFP expression by FACS analysis. The significance of the differences observed between the two conditions was determined by a Wilcoxon-Mann–Whitney analysis. NS : Not Significant. NT: untransfected cells. Number of experiments, N = 3.

Together, these results demonstrated that mRNAs packaged into retroviral particles were productively transferred into cells since they were translated into functional proteins. Despite the short half-life of luciferase, we still could observe a weak but detectable activity of supernatants collected from cells expressing luciferase, even after 9 h at room temperature (data not shown). Hence, we wanted to ensure that the luciferase activity detected in transduced cell lysates was due to mRNA transfer and their translation and not to passive luciferase transfer or pseudotransduction [32]. Therefore, we pre-treated 293FT cells with 3 μg/ml of the translation inhibitor puromycin, 1 h before transduction (Figure 7). Negative control vectors showed no activity (Figure 7, lanes 2 to 4). Cells treated with puromycin showed a significantly lower luciferase activity after retroviral mRNA transduction (Figure 7, lanes 5 to 10, black vs grey bars). The persistence of some signal upon treatment however indicated some escape from puromycin inhibition or some passive transfer of the Luciferase. We failed to elucidate the contribution of these two possibilities, since higher concentrations of puromycin were deleterious, leading to strong and rapid cell death (Data not shown). Nevertheless, these observations confirmed that the luciferase activity measured in transduced cells truly resulted from the translation of the retrovirally transferred mRNA.

## Discussion

Replication deficient retroviral vectors have been widely used in gene therapy because they promote high and long-lasting transduction efficiency [33,34]. However, because of insertional mutagenesis resulting from genomic integration, standard retroviral gene transfer should not be the preferred strategy for inducing differentiation to cure non-lethal chronic diseases in humans [35]. In recent years, various studies have shown that transfer of mRNA could fulfill the safety goals required for treating human diseases [1,2,36-38]. Besides the prominent advantage of a lack of integration, mRNA-based gene transfer may also prevent long-lasting expression of genes with pleiotropic effects and potential for cancer promotion.

Following its translocation into the cytoplasm, mRNA appears to be fairly unstable, which was considered a major hurdle to convert these molecules into therapeutic agents. Factors controlling mRNA degradation can be cis-acting or trans-acting [39]. Among the features influencing mRNA half-life, the polyA tail plays an important role in both mRNA translation and stability. The polyA tail inhibits decapping as well as degradation of mRNA [40]. Particularly in retroviruses, including murine gammaretroviruses, the efficiency of polyadenylation can be poor [41]. While it is more a concern for the safety of integrated retroviral vectors, polyadenylation might also influence the encapsidation of genomic RNA, by reducing the amount of stable transcripts during production. Interestingly, this characteristic is amenable to modification [6]. In addition, the UTR sequences of eukaryotic mRNAs have been implicated in mRNA processing, polyadenylation, stability, sub-cellular localization and translational regulation [3,17]. The 3′ UTR sequences contain signals involved in mRNA decay, one of the most-studied signals being AU-rich elements (AREs). Such sequences were described to control or promote rapid deadenylation-dependant mRNA decay [4,42]. mRNAs that contain AREs are unstable and their half-life is increased when AREs are replaced with the 3′ UTR of mRNAs showing a stable profile [43].

Considering the above data, we thought that improving mRNA stability could influence recruitment into retroviral particles. We designed a series of constructs harbouring the UTRs from genes encoding abundant cellular proteins flanking the luciferase gene. To enable the retroviral recruitment we inserted an MLV packaging signal [8,11]. We choose a minimal packaging sequence to reduce the size of the transferred mRNA as much as possible and because it was shown to be as efficient as a full length sequence [44]. However, our data suggest that the packaging step of mRNA remained susceptible to improvement, since truncated retroviral RNAs appeared to be more efficiently packaged than psi-containing mRNA (Figure 4). Among the features that could improve retroviral mRNA transfer, we thought that UTR-donating molecules from abundantly expressed proteins could be critical. However, the choice of such sequence was not trivial since, despite a high level of expression within cells, the UTRs from β-globin or β-actin did not increase mRNA stability or improve luciferase expression (data not shown). We hence focused our studies on the vimentin and NMHC UTRs. Examination of the steady-state level of the constructs at different times after blocking transcription by actinomycin D suggested that larger amounts of mRNA were detectable upon transfecting constructs containing the 5′ and 3′ UTRs from vimentin (Figure 2). However, actinomycin D strongly decreased the amount of all tested mRNA, which suggested that changing the UTR in the initial vector, for UTRs from vimentin and NMHC, did not change mRNA stability (Figure 2 C and D). Interestingly, we found that stability was not the unique characteristic to look for, because the most efficient RNAs for expression in cells were not necessarily those harbouring the most stable profile (Figure 2 and 3; for the vimentin UTRs). Importantly, in transduced cells, expression appears to result from a combination of efficient packaging and translation (Figure 4 and 6). Following transduction, it is conceivable that the stability observed for vimentin-UTR-containing mRNA could nevertheless influence expression (Figure 3A). Altogether, these data suggest that the choice of UTR sequence remains essentially empirical and that all steps, from production to transduction, should be experimentally tested. However, recent high-throughput computational analysis of whole-genome data could guide future selection processes [42].

We also generated constructs based on RNAs from a regular retroviral vector (pBullet-Luc) truncated at the 3′ end. To avoid genomic integration of the vectors, we replaced the 3′ LTR of pBullet-Luc with UTRs from vimentin or NMHC (Figure 1). The 3′ UTR from NMHC allowed the highest level of expression and packaging (Figure 5). The NMHC 3′ UTR sequence contains a cytoplasmic polyadenylation element (CPE) (+1280 to 1370; http://utrdb.ba.itb.cnr.it), with the general structure UUUUUUAU [45] which could explain the stability of the mRNA. However, although the NMHC 3′ UTR sequence appeared the most transcriptionally potent, during production, expression of the transgene was better with control vector or vectors with the vimentin 3′ UTR (Figure 3). This latter UTR contains a cis-acting element (+61 to +115) [46] able to fold into a Y-shaped secondary structure and important for vimentin mRNA function and localization in the perinuclear region of the cytoplasm. As for psi-containing mRNA, adding this 3′ UTR to mRNA led to almost equal recruitment of the mRNA into retroviral particles and equivalent luciferase expression in target cells (Figure 4 and 6). In the present study we have not addressed the question of a possible influence of a miRNA expressed in 293FT cells on the studied mRNAs. Let-7f was recently described to target NMHC [31], also the mirDB (mirDB.org) identified 25 possible miRNA for the NMHC and 17 for the vimentin, among which miR138 for example [47]. For future design of vectors, an adaptation of the UTR to the miRNA expressed within the target cell should take this into account.

Importantly, comparing truncated retroviral vectors to psi-containing mRNAs showed that, although the amount of mRNA transferred looked increased in the former (Figure 4), they promoted lower luciferase expression in transduced cells (compare each condition in Figures 4 and 6). Since they were different in their 5′UTR, we acknowledge that expression of the reporter gene could be influenced by other factors than mRNA stability. It is noteworthy that the truncated mRNA still contained the primer binding site, allowing for the formation of the strong stop during reverse transcription [48]. During strong stop formation, the RNaseH activity hydrolyses the template RNA. Thus, the 3′ truncation within the retroviral genome could generate an uncapped genomic RNA, which can be possibly less effective for translation. This was not expected since we thought that the presence of an internal ribosomal entry site encompassing the retroviral packaging signal could promote translation of those cap-deficient mRNAs [13,16]. In the pioneer study by Galla *et al.*, efficient transfer was obtained by ablating the primer binding site, which blocked the formation of the strong stop, the first molecular species synthesized during reverse transcription [14,48]. Accordingly, translation of truncated mRNAs will certainly take advantage of the deletion of the primer-binding site. A highly context-dependent translation of the retroviral genome was recently described [49], suggesting that further studies addressing the retroviral UTR sequence could help in defining the most efficient context for mRNA translation upon transfer. Looking for further improvement, it is noteworthy that the stability of synthetic mRNA could be improved by a combination of nucleoside optimisation and the choice of the UTR content [50].

## Conclusions

In summary, using a retroviral mobilization system, we have been able to efficiently recruit various mRNAs into retroviral particles. Furthermore, viral transfer of such mRNAs into target cells led to a transgene-induced biological effect, which validates the development of this approach for future use in cell re-programming. At present, the system appears more suitable for specific, transient, low-level expression of a transgene. In the future, retroviral RNA-mobilisation vectors may be further improved by thoroughly evaluating other combinations of UTRs, use more stable cDNA coding sequences [50] and the addition of constitutive transport elements, which increase the amount of cytoplasmic packageable RNA [51]. All these adaptations should increase the mRNA level in the producing cell and thereby extend the applications of retroviral mRNA transfer.

## Methods

### Plasmid construction

PCR and specific primers were used to obtain the entire luciferase Firefly sequence (Table 1) which was cloned into the pcDNA3.1(−) vector (Invitrogen) at the HindIII and KpnI sites to generate pcDNA-Luciferase. PCR was used with specific primers to obtain the 5′ and 3′ UTRs from human vimentin and non-muscular myosin heavy chain (NMHC, MYH9) (beginning 3′ of the stop codon and extending to the first polyA site) (Table 1), which were subcloned into the pGemTeasy vector (Promega). To generate pcDNA-Luciferase-5′UTR plasmids, the 5′ UTR sequence was cloned into the NheI-HindIII site in the pcDNA-Luciferase vector, and to generate pcDNA-Luciferase-3′UTR plasmids, the 3′ UTR sequence was cloned into the KpnI-NotI site in the pcDNA luciferase vector.

**Table 1:** Primers for PCR amplification

**Target**	**Primer**	**Primer Sequence (5′-3′)**	**Sequence Length**
Luciferase	Luciferase-S	5′-TCg**AAgCTT**ACCATggAAgACgCCA -3′	1677 bp
	Luciferase-AS	5′-gAAT**ggTACC**TTACAATTTggAC -3′	
Vimentin	Vimentin-5UTR-AS	5′ ATCg**gCTAgC**gCgTCCCCgCgCCAg -3′	144 bp
	Vimentin-5UTR-AS	5′-gAA**TAAgCTT**ggCTgCggAgggT -3′	
	Vimentin-3UTR-S	5′-ATCg**ggTACC**AAATTgCACACAC -3′	325 bp
	Vimentin-3UTR-AS	5′-gAAT**gCggCCgC**gAAgCAgAACC -3′	
Myosin	Myosin-5UTR-S	5′-TCg**gCTAgC**gAAggCTAAgCA -3′	154 bp
	Myosin-5UTR-AS	5′-AATT**AAgCTT**ACCTgAACCTg -3′	
	Myosin-3UTR-S	5′-ATCg**ggTACC**gCCTCTTCTCCTg -3′	1389 bp
	Myosin-3UTR-AS	5′-gAAT**gCggCCgCg**TgATgCTCAg -3′	
MLV Packaging signal	psi-S	5′ = AAAgT**ggTACC**gggAggTAAgCT-3′	322 bp
	psi-AS	5′-gCCTT**ggTACC**gAACTgTTTTAg-3′	

Sequence amplified for modifying the vectors (restriction sites used allowing the cloning are in bold).

To generate pcDNA-Luciferase-psi-UTR, psi derived from the MLV virus (+206; +512) was amplified from pLNCX with use of specific primers (Table 1) and cloned into the KpnI site 3′ of the luciferase gene in pcDNA-Luc and pcDNA-Luc-UTR plasmids (Figure 1).

To generate truncated mRNA, the pBullet-Luc plasmid was digested with SacI and ClaI or SacI and EcoRV. The fragment containing the 5′ LTR, psi and the luciferase gene was cloned into pcDNA-Luc and pcDNA-Luc-UTR. The resulting plasmids containing optimal packaging sequences were designated pCMV-5′LTR-psi-Luc and pCMV-5′LTR-psi-Luc-3′UTR (Figure 1).

### Cells

The 293FT cells (derived from the clone ATCC CRL-11268) were maintained in Dulbecco’s modified Eagle’s medium (DMEM) (GlutaMAX) supplemented with pyruvate, penicillin (100 U/ml), streptomycin (100 μg/ml), 10% heat inactivated fetal calf serum and 1% non-essential amino acids.

### Assessment of mRNA stability

293FT cells were seeded at 200,000 cells per well into 24-well plates and grown overnight at 37°C, then transfected with 200 ng of each construct using the Fugene 6 transfection reagent (Roche). After 10 h, cells were washed with phosphate buffered saline (PBS), and fresh medium was added. After 24 h, actinomycin D (1 μg/ml, Sigma-Aldrich) was added into each well. At 2 to 4 days post-transfection, cells were washed 3 times with PBS and harvested; total RNA was extracted by use of the Nucleospin RNAII kit (Macherey Nagel). Cells were also harvested on the day of actinomycin D treatment. To eliminate plasmid DNA contamination, RNA samples were treated once with TURBO DNase (2 U/μl, Ambion) and purified by use of the RNA clean-up kit (Macherey Nagel). To confirm the absence of plasmid DNA contamination, an end-point PCR was performed on each RNA samples, negative results allowed us to proceed further the experiment (not shown). First-strand cDNA synthesis involved the Superscript First-Strand II Synthesis system (Invitrogen) with 300 ng total RNA and random hexamers. Quantitative PCR involved the Light Cycler-480-II system (Roche). PCR amplification of the reaction mixture (2 μl of reverse transcriptase product, 1X SYBR Green I master mix [Roche], 10 μM each primer and RNase-free water to a final volume of 20 μl) involved 5 min pre-amplification at 95°C, 45 cycles of 10 seconds at 60°C and 10 seconds at 72°C, then melting curve and cooling steps. PCR amplification involved use of the primer sequences for luciferase, forward, 5′-CAACTgCATAAggCTATgAAgAgA-3′ and reverse, 5′-ATTTGTATTCAgCCCATATCgTTT-3′ (Makawa *et al.*, 2008); and β-actin, forward 5′-CgCACCACTggCATTgTCAT-3′ and reverse, 5′-TTCTCCTgATgTCACgCAC-3′), as a normalization control. Data are expressed as the mean of 2 experiments performed in duplicate.

293FT cells were seeded at 30,000 cells per well into 96-well plates and grown overnight at 37°C. Fugene 6 transfection reagent (Roche) was used to co-transfect cells with 50 ng pCDNA-Luc-psi, pCDNA-Luc-psi-UTR, pCMV-5′LTR-psi-Luc or pCMV-5′LTR-psi-Luc-UTR and 5 ng pGL4.74 (Promega) containing Renilla luciferase to normalize Firefly luciferase. At 24 h post-transfection, actinomycin D (1 μg/ml) was added to each well. At 2 to 4 days post-transfection, Firefly luciferase expression was measured by Dual-Glo luciferase assay (Promega) with normalization to Renilla expression. Activity for each construction was compared to that for pcDNA-Luc-psi alone.

### Cell transfection and retroviral particle production

293FT cells were plated at 4.2×10^6^ cells per plate into a 10-cm dish and grown overnight. Fugene 6 transfection reagent was used to transfect cells with 1 μg pMNGag-Pol, 1 μg of pCMV-Env encoding an amphotropic envelope, 80 ng of a reporter plasmid for normalization, pEGFPC1, and 3.22 10^-13^ mole of the different test constructs, pcDNA-Luc-psi-UTR or pCMV-5′LTR-psi-Luc-UTR or control constructs lacking the retroviral psi, pcDNA-Luc. After 10 h, the medium was removed and cells were washed with PBS, then fresh medium was added. After 48 and 72 h, retroviral particles containing supernatants were harvested, filtered through a 0.45-μm-pore filter (Millipore) and incubated for 3 h at 4°C before 293FT transduction. pBullet-Luc, pMNGagPol and pCMV-Ampho were used as positive controls. pBullet-Luc, pMNGagPol or pBullet-Luc, pCMV-Env without pMNGagPol were used as negative controls.

### Quantitative real-time RT-PCR

To evaluate mRNA transfer, 293FT cells (230,000 cells/well in 24-well plates) were transduced with 500 μl supernatants containing retroviral particles. Transduction was improved by the addition of polybrene (5 μg/ml) and centrifugation for 1 h at 1900 rpm and 37°C. After 3 and 6 h, medium was removed and cells were washed 3 times with PBS. Then cells were harvested and total RNA was extracted by use of the Nucleospin RNAII kit (Macherey Nagel). To eliminate plasmid DNA contamination, total RNA was treated with Turbo DNAse (2 U/μl; Ambion) and purified using Nucleospin RNA clean-up kit (Macherey Nagel) according to the manufacturer’s instructions. The efficiency of this treatment was verified using end-point PCR, negative results allowed us to proceed further the experiment (Not shown). cDNA synthesis was performed as above. In total, 2 μl cDNAs were used for quantitative RT-PCR (RT-qPCR) with SYBR Green I Master and LightCycler-480-II (Roche). RT-qPCR was carried out as above.

To assess mRNA mobilisation into retroviral particles, RNA was extracted from 140 μl supernatants containing retroviral particles by use of the QIAamp viral RNA Mini-kit (Qiagen). After extraction, RNA was treated with TURBO DNAse (2 U/μl; Ambion). Then, DNAse was removed using the Nucleospin RNA clean-up kit (Macherey-Nagel) according to the manufacturer’s instructions. An end-point PCR was performed to verify that there was no plasmid DNA contamination in the RNA treated with DNAse (Not shown). In total, 4 μl RNA treated with TURBO DNAse (2 U/μl) was used for cDNA synthesis with the Superscript First-strand Synthesis System (Invitrogen). Luciferase RNA copies were quantified by qRT-PCR with luciferase primers and the SYBR Green I master Kit (Roche) as above. The RNA copy number was calculated by an external standard curve of serial dilution of pBullet-Luc plasmid.

### Luciferase assay

On the day before transduction, 60,000 cells were seeded in 96-well plates. On the day of transduction, 100 μl supernatants containing retroviral particles were applied to cells with or without 3 μg/ml puromycin. This translation inhibitor was added 1 h before transduction. Transduction was assisted by the addition of polybrene (5 μg/ml) and centrifugation for 1 h at 1900 rpm and 37°C. At 3 and 6 h post-transduction, luciferase expression was determined by use of the Bright-Glo luciferase assay (Promega) and a Centro LB 960 luminometer (Berthold Technologies). Each assay was performed in triplicate; all experiments were repeated 3 times.

### Statistical analysis

Statistical analyses were performed on 3 independent experiments. Each experiment was carried out in triplicate. Data are expressed as mean ± SD; Wilcoxon–Mann–Whitney, one sample *t*-test or one sample *t*-test were used to analyse the data.

## Competing interest

The authors have no conflict of interest to declare.

## Authors’ contributions

AP designed and performed the experiments and contributed to manuscript writing. LS contributed to manuscript writing. JCP initiated the project, supervised the design and the experiments, and wrote the manuscript. All authors read and approved the final manuscript.
